# CHD7 Maintains Neural Stem Cell Quiescence and Prevents Premature Stem Cell Depletion in the Adult Hippocampus

**DOI:** 10.1002/stem.1822

**Published:** 2014-12-18

**Authors:** Kieran M. Jones, Nemanja Sarić, John P. Russell, Cynthia L. Andoniadou, Peter J. Scambler, M. Albert Basson

**Affiliations:** ^1^ King's College London, Department of Craniofacial Development and Stem Cell Biology Guy's Hospital Tower Wing London UK; ^2^ Molecular Medicine Unit UCL Institute of Child Health London UK; ^3^ King's College London, MRC Centre for Developmental Neurobiology London UK

**Keywords:** CHD7, Chromatin remodeling, Adult neurogenesis, Neural stem cell, Quiescence, Maintenance, Hippocampus, Subgranular zone, Mouse

## Abstract

Neural stem/progenitor cells (NSCs) in the hippocampus produce new neurons throughout adult life. NSCs are maintained in a state of reversible quiescence and the failure to maintain the quiescent state can result in the premature depletion of the stem cell pool. The epigenetic mechanisms that maintain this quiescent state have not been identified. Using an inducible knockout mouse model, we show that the chromatin remodeling factor chromodomain–helicase‐DNA‐binding protein 7 (CHD7) is essential for maintaining NSC quiescence. CHD7 inactivation in adult NSCs results in a loss of stem cell quiescence in the hippocampus, a transient increase in cell divisions, followed by a significant decline in neurogenesis. This loss of NSC quiescence is associated with the premature loss of NSCs in middle‐aged mice. We find that CHD7 represses the transcription of several positive regulators of cell cycle progression and is required for full induction of the Notch target gene *Hes5* in quiescent NSCs. These findings directly link CHD7 to pathways involved in NSC quiescence and identify the first chromatin‐remodeling factor with a role in NSC quiescence and maintenance. As CHD7 haplo‐insufficiency is associated with a range of cognitive disabilities in CHARGE syndrome, our observations may have implications for understanding the basis of these deficits. Stem Cells *2015;33:196–210*

## Introduction

New neurons are continuously generated from neural stem/progenitor cells (NSCs) that reside in discrete regions of the adult mammalian forebrain 1, 2, 3, 4, 5. NSCs located in the subgranular zone (SGZ) of the dentate gyrus (DG) divide infrequently and produce new neurons after transiting through a rapidly proliferating transit amplifying progenitor stage 1, 5. Newborn granule cells produced in the DG are capable of integrating into existing circuitry and play an important role in certain types of hippocampus‐dependent learning tasks and memory formation 6, 7, 8, 9.

Quiescence is a reversible state of growth arrest crucial for the preservation of somatic stem cell number and function 10, 11, 12, 13, 14, 15. Failure to maintain quiescence can result in the accelerated conversion of stem cells to progenitors and depletion of the stem cell pool 10, 15. For example, deletion of RBPJ (recombination signal binding protein for immunoglobulin kappa J region), the downstream effector of Notch signaling, or the Notch1 receptor itself, leads to a loss of NSC quiescence, a rapid depletion of the stem cell pool, and an irreversible deficit in neurogenesis 16, 17, 18. Similarly, loss of the cyclin‐dependent kinase inhibitors p21 or p57 and cell cycle inhibitor Btg1, all cause an increase in NSC proliferation and the eventual depletion of the stem cell pool that has been ascribed to proliferative exhaustion of the quiescent NSC pool 13, 19, 20.

Chromatin remodeling factors can alter the accessibility of DNA to the transcriptional machinery through the eviction of nucleosomes, initiating nucleosome sliding, or by facilitating DNA looping 21. Chromatin remodeling factors play important roles in stem cell function and daughter cell differentiation; however, whether they have a role in regulating NSC quiescence is not known 22, 23. Chromodomain–helicase‐DNA‐binding protein 7 (CHD7) is a member of the CHD family of ATP‐dependent chromatin‐remodeling enzymes 24. Recent studies have identified a role for CHD7 in embryonic NSC function 25, and adult NSC differentiation 26. Furthermore, CHD7 has been shown to interact with the NSC regulator SOX2 to regulate target gene transcription in fetal neural stem cells 27.

Using genetic mouse models and an in vitro quiescence assay, we have found that CHD7 deletion in NSCs results in a loss of stem cell quiescence and a transient increase in immature neuron formation, before an eventual decline in neurogenesis. Interestingly, loss of stem cell quiescence after deletion of CHD7 in adult NSCs leads to a large decrease in the number of NSCs in the hippocampus of middle‐aged mice as the stem cell pool is depleted. We find that loss of CHD7 results in the misregulation of genes involved in cell cycle progression and Notch signaling, directly linking CHD7 to pathways with established roles in NSC quiescence.

## Materials and Methods

### Animals

Adult CD1 mice were obtained from Charles River, U.K. *GLAST:: CreER^T^^2^* 28, *Nestin‐Cre* 29, and *Rosa‐EYFP (RYFP)* 30 mice have been described previously. Mice with *Chd7* exon 3 flanked by LoxP sites (*Chd7^tm^^2^^a(^^EUCOMM^^)Wtsi^*) were generated from an ES clone (Emma ID, EM:04817 Cell line, EPD0019_1_D07), hereinafter designated the *Chd7^f^* allele. Following Flpe‐mediated removal of the β‐geo cassette by crossing to Tg(ACTFLPe)9205Dym, the conditional allele was validated using Tg(ACTB‐cre)2Mrt to create and phenocopy a constitutively null allele (P.J. Scambler, unpublished observations).

All experiments were initiated in adult mice between 10 and 12 weeks of age. All animal procedures were approved by the U.K. Home Office. *GLAST::CreER^T^^2^;Chd7^f/f^* or *GLAST:: CreER^T^^2^;Chd7^f/f^;RYFP/+* mice were used to delete *Chd7* from adult NSCs upon tamoxifen (TAM) administration. *Chd7^f/f^* or *Chd7^f/f^;RYFP/+* mice were used as controls throughout. We confirmed that TAM‐treated *GLAST::CreER^T^^2^;RYFP/+* mice exhibited identical phenotypes to other control mice, ruling out any effects from Cre toxicity or nonspecific effects of TAM.

### In Vitro Cell Culture and FACS Isolation

Fetal‐derived NSCs from a mating between *Nestin‐Cre;Chd7^f/+^* and *Chd7^f/f^* mice were isolated from the cortex and striatum of E16.5 embryos. After genotyping, cultures from *Nestin‐Cre;Chd7^f/f^* embryos and Cre‐negative embryos were established. Cell growth and quiescence conditions were as described previously 31, 32, 33, 34, 35. For quiescence and proliferation analyses, 3 × 10^4^ cells were plated on eight‐well glass chamberslides (Nunc, Thermo Scientific, MA, http://www.thermoscientific.com) precoated with 0.01% poly‐l‐ornithine (Sigma‐Aldrich, MO, http://www.sigmaaldrich.com) overnight followed by 10 µg/ml laminin (Sigma‐Aldrich) for 1 hour at 37°C.

*pcDNA3.1* empty vector control plasmid and *pcDNA::hChd7* plasmid constructs (kindly provided by Dr. J. Wysocka) 36, and *pCLIG* empty vector control plasmid and *pCLIG*::*rHes5* plasmid constructs (kind gifts from Dr. R Kageyama) 37, were transfected into fetal‐derived NSCs using Amaxa nucleofection (Amaxa Biosystems, Switzerland, http://www.lonza.com/research/). Briefly, 10 µg of plasmid was transfected into 8 × 10^6^ NSCs using the Primary Neuron Nucleofector kit and program A‐033, according to manufacturer's recommendations. For experiments involving *pcDNA::hChd7* constructs, 24 hours after nucleofection, one sample of cells were trypsinized and taken into Trizol for RNA isolation. A second sample was transferred to quiescence conditions for 4 days before RNA was extracted. For experiments involving *pCLIG*::*rHes5* constructs, cells were put into quiescence conditions 24 hours after nucleofection and analyzed 24 hours later.

For FACS isolation, NSCs from the hippocampus of four adult *GLAST::CreER^T^^2^;RYFP/+* and *GLAST::CreER^T^^2^;Chd7^f/f^;RYFP/+* mice were dissociated as described previously 38. Live (DAPI‐negative), YFP‐negative, and YFP‐positive cells were sorted directly into Trizol (Life Technologies; http://www.lifetechnologies.com) using a FACS Aria II (BD Biosciences, U.K., http://www.bdbiosciences.com).

### Western Blot

Total cell lysates were prepared by lysing 1 × 10^6^ cells in 100 µl 8 M urea, 1% CHAPS, 50 mM TRIS pH7.9 followed by the removal of DNA by centrifugation. Proteins (20 µg per lane) were resolved on a 3–7% Tris‐acetate gel (Life Technologies) and transferred to a nitrocellulose membrane (Life Technologies). After blocking with 0.5% nonfat milk powder in TBS with 0.5% Tween 20 (TBST), the membrane was incubated with primary antibodies (αCHD7, Abcam, U.K., http://www.abcam.com, ab31824, 1/2000; αCPSF100, Bethyl, TX, http://www.bethyl.com, A301–581A, 1/1000) in 5% BSA, TBST overnight at 4°C. After washing and incubation with horse radish peroxidase (HRP)‐conjugated secondary antibody (Millipore, U.K., http://www.emdmillipore.com) for 1 hour at room temperature, HRP conjugates were detected using Clarity Western ECL reagent (Bio‐rad, U.K., http://www.bio-rad.com). Gels were visualized on a Bio‐Rad gel doc system and images prepared using Adobe Photoshop.

### RNA Isolation and Quantitative RT‐PCR Analysis

RNA extraction from approximately 1 × 10^6^ fetal‐derived NSCs, a minimum of 1 × 10^3^ FACS‐isolated cells, and the microdissected DG of 2–3 animals per condition was performed with Trizol (Life Technologies) according to the manufacturer's suggested modifications, by the addition of ultrapure glycogen (Life Technologies). First‐strand complementary DNA was synthesized from 200 ng of RNA using the Precision Nanoscript Reverse Transcriptase kit (PrimerDesign Ltd., U.K., http://www.primerdesign.co.uk) according to manufacturer's recommendations. Real‐time quantitative polymerase chain reaction (RT‐qPCR) was performed on a Stratagene Mx3000p Real Time PCR machine (Agilent Technologies, CA, http://www.home.agilent.com), with Precision 2× RT‐PCR MasterMix (PrimerDesign Ltd.) using primers against *Chd7*, h*Chd7* (which may recognize mouse *Chd7*), *Hes5,* r*Hes5* (which may recognize mouse *Hes5*), *Pax6* (Designed by PrimerDesign Ltd.)*, Ccnb1, Ccnd1, Ccnd2, Ccne1, Cdk1, Cdk2, Btg1, Mtor*, *Nestin*, and *Gapdh* as a normalizing control to give ΔΔCq values relative to wild‐type samples. Primer sequences are shown in Supporting Information Table 1.

### Forebrain Processing

Adult mice were given a lethal dose of Euthanal (pentobaribital sodium, Merial, U.K., http://www.merial.co.uk) and perfused with ice‐cold PBS followed by ice‐cold 4% PFA. Brains were dissected and further fixed in 4% PFA overnight at 4°C. For immunohistochemistry, fixed brains were cryoprotected in 15% sucrose and 7.5% gelatine and frozen in methylbutane (Sigma‐Aldrich) in dry ice.

### Immunohistochemistry and Immunocytochemistry

Coronal brain sections (20‐µm‐thick) were prepared using a Cryotome (Bright Instrument Co Ltd., U.K., http://www.brightinstruments.com) on Superfrost+ slides. Slides were incubated in PBS at 37°C for 40 minutes to remove gelatine and then post‐fixed in 4% PFA for 10 minutes. For 5‐bromodeoxyuridine (BrdU), CHD7, MCM2, and S100β detection, antigen‐retrieval was performed by incubating slides with 2N HCl for 17 minutes at 37°C. Acid was neutralized by incubating slides in 0.1 M borate buffer, pH 8.5. Sections were permeabilized by washes with 0.1% Triton X (Sigma) in PBS (PBSTx). Sections were blocked in 10% heat‐inactivated goat serum (GS) for 1 hour before incubation with primary antibody diluted in 5% GS in PBSTx overnight at 4°C. The antibodies used were as follows: rat anti‐BrdU (1/50, Abcam), mouse anti‐Ki67 (1/50, Abcam), mouse anti‐SOX2 (1/800, Abcam), rabbit anti‐CHD7 (1/80, Abcam), rabbit anti‐glial fibrillary acidic protein (GFAP) (1/500, Sigma), chicken anti‐YFP (1/200, Abcam), rabbit anti‐S100β (1/100, Abcam) rabbit anti‐cleaved caspase‐3 (1/200, Cell Signaling) mouse anti‐GFAP (1/1000, Abcam), mouse anti‐BM28 (MCM2; 1/400, BD Biosciences), mouse anti‐ASCL1 (1/200, BD Biosciences), rabbit anti‐DCX (1/500, Abcam), mouse anti‐NeuN (1/100, Chemicon, CA, http://www.chemicon.com). Sections were incubated with Alexa‐Fluor‐labeled secondary antibodies diluted at 1/200 (Life Technologies) and DAPI at 1/5000 in 5% GS in PBSTx for 1 hour before mounting on to coverslips with Citifluor (Citifluor Ltd., U.K., http://www.citifluor.com). For double immunofluorescence of CHD7 with GFAP, sections were degelatinized and permeabilized as above, and blocked for 1 hour in blocking buffer containing 0.1 M Tris‐HCl pH7.5, 0.15 M NaCl, and 0.5% blocking reagent (Perkin Elmer, U.K., http://www.perkinelmer.co.uk). Incubation with primary antibody against CHD7 (Abcam) for 1 hour at room temperature at 1/700 in TNT buffer (0.1 M Tris‐HCl, 0.15 M NaCl, and 0.05% Tween), was followed by washes and incubation with goat anti‐rabbit biotin (Abcam) at 1/300 in TNT for 1 hour, anti‐GFAP (DAKO) 1/200 in TNT for 1 hour, goat anti‐rabbit‐488 (Abcam) 1/350 in TNT for 1 hour for detection of anti‐GFAP, ABC reagent (Vector Laboratories Ltd., U.K., http://www.vectorlabs.co.uk) for 30 minutes at room temperature, and finally, Cyanine 5 Tyramide signal amplification reagent (Perkin Elmer, MA, http://www.perkinelmer.com) for 10 minutes to amplify the CHD7 signal, with adequate washes in TNT between all steps. Slides were mounted in Vectashield containing DAPI (Vector Laboratories Ltd).

Cells on chamber slides were fixed with 4% PFA for 10 minutes before premeabilization in PBSTx and blocking in 10% GS in PBSTx for 1 hour. Immunostaining was then performed as above.

Fluorescent images were captured on a confocal laser‐scanning microscope (Leica SP5, Germany, http://www.leica.co.uk) using a 63× glycerol‐immersion objective, or an 80i compound microscope (Nikon Ltd., U.K., http://www.nikon.co.uk). Images were processed using NIS Elements Viewer 4.0 or LAS AF Lite and Adobe Photoshop CS5 software.

### Analysis of NSCs and Their Progeny

For the analysis of cells expressing CHD7, a minimum of 100 CHD7^+^ cells were analyzed across 24 hippocampal sections at least 60 µm apart from three wild type mice by a max projection of a 20‐µm Z‐stack in 0.5 µm steps on a confocal laser‐scanning microscope. For the quantification of the number of CHD7^+^, DCX^+^, BrdU^+^, GFP^+^, NeuN^+^, S100β^+^, cleaved caspase‐3^+^, SOX2^+^, or GFAP^+^ cells a minimum of 52 DG sections at least 60 µm apart were quantified across a minimum of three animals per condition. For the analysis of the number of proliferating NSCs, a minimum of 200 BrdU^+^ cells were analyzed by confocal microscopy from at least 60 DG sections across at least four animals per condition. For the analysis of in vitro proliferating cells, automated particle analysis with ImageJ software (National Institute of Health, http://rsbweb.nih.gov/ij/) was performed on a minimum of 4000 cells per culture condition. All counts were performed blind.

### In Vivo Activation of Cre Recombinase and Cell Division Analysis

Adult mice were given one intraperitoneal (I.P.) injection of 80 mg/kg (typically 120 µl of 20 mg/ml) TAM (Sigma‐Aldrich) diluted in corn oil (Sigma‐Aldrich) daily for 5 days. To analyze SGZ cell proliferation, animals were given one I.P. injection of BrdU (Sigma‐Aldrich) dissolved in isotonic saline (0.9% NaCl) at a concentration of 75 mg/kg, 24 hours before sacrifice 39.

### Statistical Analysis

All data are represented as mean ± SEM; *, *p* < 0.05; **, *p* < 0.01; ***, *p* < 0.001, Student's *t*‐test.

## Results

### CHD7 Is Expressed in Hippocampal NSCs

**Figure 1:**
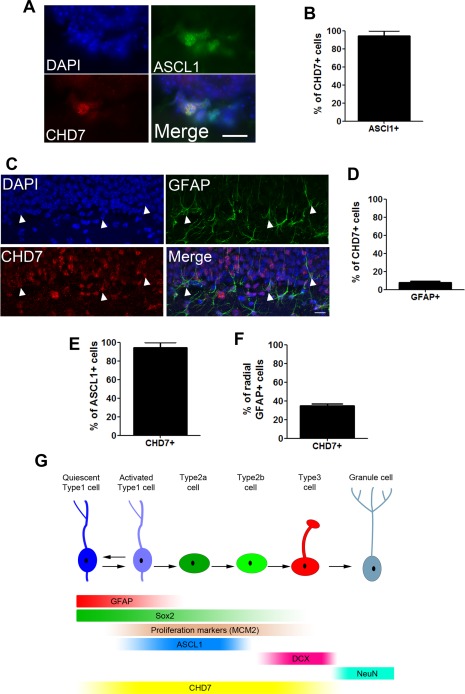
Chromodomain–helicase‐DNA‐binding protein 7 (CHD7) is expressed in hippocampal neural stem cells and progenitors. **(A):** Coronal adult mouse dentate gyrus (DG) section showing the subgranular zone (SGZ) immunostained with anti‐CHD7 and anti‐ASCL1 antibodies and DAPI. Scale bar = 20 µm. **(B):** Quantification of the percentage of CHD7^+^ cells that express ASCL1. **(C):** Coronal adult mouse DG section showing the SGZ immunostained with anti‐CHD7 and anti‐glial fibrillary acidic protein (GFAP) antibodies and DAPI. White arrow heads show SGZ CHD7^+^ cells with a radial GFAP process. Scale bar = 20 µm. **(D):** Quantification of the percentage of CHD7^+^ cells that also display a radial GFAP^+^ process. **(E, F):** Quantification of the percentage of ASCL1^+^ **(E)** and radial GFAP^+^ cells **(F)** that express CHD7. *n* = 100–150 cells from 2 to 3 animals. **(G):** Schematic illustration of CHD7 expression in the adult hippocampal neurogenic niche. Abbreviations: ASCL1, achaete‐scute complex homolog 1; CHD7, chromodomain–helicase‐DNA‐binding protein 7; DAPI, 4′,6‐diamidino‐2‐phenylindole; DCX, doublecortin; GFAP, glial fibrillary acidic protein; MCM2, minichromosome maintenance deficient 2 mitotin.

To determine whether CHD7 is present in NSCs in the adult hippocampus, cryosections were immunostained with antibodies to CHD7, GFAP (to identify radial NSCs) and ASCL1 (achaete‐scute complex homolog 1, to identify neuronal progenitors) 40, 41, 42. The majority of CHD7^+^ cells were ASCL1^+^ (Fig. 1A, 1B). Importantly, a small, but significant fraction (8%) of CHD7^+^ cells were GFAP^+^ radial NSCs in the SGZ (Fig. 1C, 1D), implying a role for CHD7 in hippocampal NSCs. An analysis of CHD7 expression in different cellular subsets confirmed that 95% of ASCL1^+^ cells expressed CHD7 (Fig. 1E). Taken together with data from Feng et al. who showed that the majority of CHD7 is present in rapidly dividing cells 26, it appears that CHD7 expression might be upregulated in type 1 NSCs as they exit the quiescent state and that high CHD7 expression marks most progenitors as they progress through a highly proliferative type 2 ASCL1^+^ transient amplifying state towards neurons (Fig. 1G). Interestingly, a careful analysis of GFAP^+^ radial NSCs in the SGZ indicated that 35% of type 1 cells were positive for CHD7 (Fig. 1F). As only a small fraction (1–4%) of these cells actively divide (see Fig. 6D, 6E; 19), these observations indicate that CHD7 is expressed in a proportion of NSCs that would include quiescent and activated subsets (Fig. 1G).

### Deletion of *Chd7* in NSCs

**Figure 2:**
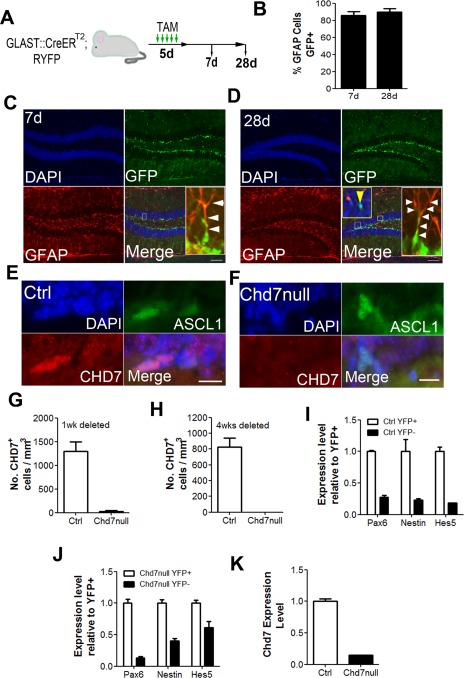
Deletion of *Chd7* from GLAST and Nestin‐expressing progenitors. **(A):** Schematic of the experimental strategy to induce recombination and YFP expression in subgranular zone (SGZ) neural stem/progenitor cells (NSCs). Adult *GLAST::CreER^T2^;RYFP/+* mice were given one injection of 80 mg/kg tamoxifen (TAM) a day for 5 days and analyzed either 7 days or 28 days after TAM injection. **(B):** Quantification of the percentage of radial GFAP^+^ cells that were GFP^+^ in the SGZ 7 days (7d) and 28 days (28d) after TAM injection. At least 90 GFAP^+^ cells were counted. *n* = 2 animals. **(C, D):** Representative image of a coronal section of the dentate gyrus of *GLAST::CreER^T2^;RYFP/+* mice 7 days **(C)** or 28 days **(D)** after TAM injection stained with antibodies against green fluorescent protein (GFP) and glial fibrillary acidic protein (GFAP). GFP antibodies recognize YFP protein. White arrow heads show GFAP^+^GFP^+^ astrocytic processes. Yellow arrow head shows a GFAP^‐^GFP^+^ cell in the granular layer in **(D)**. Note the significant overlap between GFAP and GFP. **(E, F):** Representative image of a section of the SGZ stained with antibodies raised against CHD7 and ASCL1 in control **(E)** and Chd7null **(F)** adult brains 4 weeks after TAM administration. Sections were stained with antibodies against ASCL1 to identify the NSC population that normally express high levels of CHD7. **(G, H):** Quantification of the number of CHD7^+^ cells in the SGZ of control (Ctrl) and Chd7null mice 1 week **(G)** and 4 weeks **(H)** after TAM injection. *n* = 2–4 animals per condition. **(I, J):** RT‐qPCR quantification of *Pax6* expression in YFP^‐^ and YFP^+^ live (DAPI‐negative) cells FACS‐isolated from microdissected dentate gyrus (DG) of *GLAST::CreER^T2^;RYFP/+* (control; Ctrl; **I**) and *GLAST::CreER^T2^;Chd7^f/f^;RYFP/+* (Chd7null; **J**) mice 7 days after TAM injection. A minimum of 1000 cells from 4 animals per condition were isolated. **(K):** RT‐qPCR quantification of *Chd7* expression in FACS‐isolated YFP^+^ control and Chd7null NSCs. Data from **(I–K)** are from four reactions. All data represented as mean ± SEM. Abbreviations: ASCL1, achaete‐scute complex homolog 1; CHD7, chromodomain–helicase‐DNA‐binding protein 7; DAPI, 4′,6‐diamidino‐2‐phenylindole; GFAP, glial fibrillary acidic protein; GFP, green fluorescent protein; RFYP, *Rosa‐EYFP*; TAM, tamoxifen; YFP, yellow fluorescent protein.

To investigate the function of CHD7 in the adult hippocampus, we utilized the *GLAST::CreER^T^^2^* mouse line to delete *Chd7* in NSCs in the adult brain 28. We first determined the recombination efficiency in the DG by crossing the *GLAST:: CreER^T^^2^* line to a *RYFP* reporter line to generate *GLAST:: CreER^T^^2^;RYFP/+* mice. Adult *GLAST::CreER^T^^2^;RYFP/+* mice were analyzed 7 and 28 days after tamoxifen (TAM) injection (Fig. 2A). Seven days after TAM administration, 78–91% of radial GFAP^+^ cells were YFP^+^, showing efficient recombination in NSCs (Fig. 2B, 2C). Twenty‐eight days after the last tamoxifen injection, YFP^+^ cells in the granular layer of the DG were visible, representing newborn neurons derived from Cre‐recombined NSCs (Fig. 2D). Furthermore, at this time point 86–94% of radial GFAP^+^ cells were still YFP^+^ (Fig. 2B, 2D), indicating that quiescent NSCs that have undergone Cre recombination are maintained in the niche. Importantly, no YFP^+^ cells were observed in mice not injected with TAM, indicating no leaky Cre or YFP expression (data not shown). Therefore, the *GLAST::CreER^T^^2^* line allows for efficient recombination in hippocampal NSCs.

To delete *Chd7* specifically from adult NSCs, *GLAST::CreER^T^^2^;Chd7^f/f^* mice were generated. To test the efficiency of *Chd7* deletion, sections from the DG of *GLAST::CreER^T^^2^;Chd7^f/f^* (Chd7null) and Cre‐negative control mice (control) were stained with antibodies against CHD7 (Fig. 2E, 2F). Fewer than 3% of the number of CHD7‐positive cells present in controls was detected in the DG of Chd7null mice, indicating highly efficient deletion of *Chd7* in vivo (Fig. 2G, 2H).

To further confirm the expression and efficient deletion of CHD7 in adult NSCs, *GLAST::CreER^T^^2^;RYFP/+* (control) and *GLAST::CreER^T^^2^;Chd7^f/f^;RYFP/+* (Chd7null) mice were injected with TAM and brains were collected 7 days after the last injection. Live (DAPI‐negative), YFP‐positive, and YFP‐negative cells were FACS‐isolated from the hippocampus and RNA was extracted. YFP^+^ cells from both control and Chd7null mice expressed high levels of the NSC markers *Pax6* 42, *Nestin*, and *Hes5* 43 compared with YFP^‐^ cells (Fig. 2I, 2J). Correspondingly, *Chd7* expression was markedly reduced in YFP^+^ sorted cells from Chd7null mice (Fig. 2K). Collectively, these data demonstrate that CHD7 is expressed by adult hippocampal NSCs and that CHD7 can be ablated effectively in these cells in the adult hippocampus.

### CHD7 Is Required for Neural Stem Cell Quiescence

**Figure 3:**
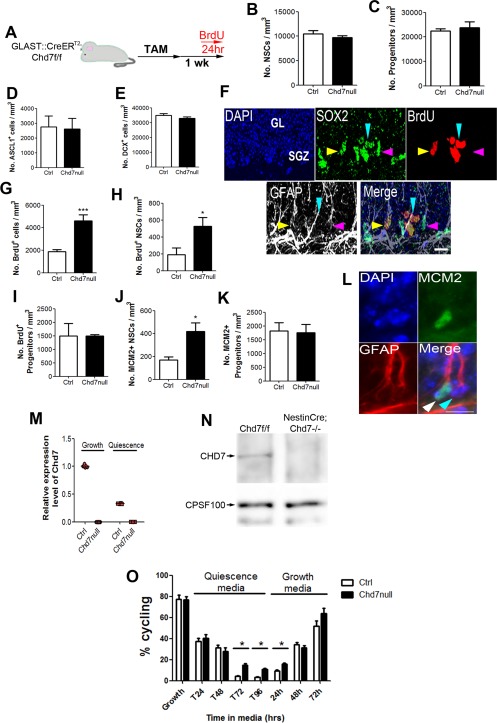
Chromodomain–helicase‐DNA‐binding protein 7 (CHD7) regulates neural stem/progenitor cell (NSC) quiescence. **(A):** Schematic diagram of the experimental strategy to delete *Chd7* from adult NSCs. Six days after tamoxifen (TAM) administration 5‐bromodeoxyuridine (BrdU) was injected and brains collected 24 hours later. **(B–D):** Quantification of the number of radial GFAP^+^SOX2^+^ NSCs (B), GFAP^‐^SOX2^+^ progenitor cells, and ASCL1^+^ stem/progenitor cells (D) in the subgranular zone (SGZ) of Chd7null and control (Ctrl) mice 1 week after TAM injection. **(E):** Quantification of the number of DCX^+^ cells in the dentate gyrus (DG) of Chd7null and control (Ctrl) mice 1 week after TAM injection. **(F):** Representative image of a section of the DG stained with antibodies against BrdU, SOX2, and glial fibrillary acidic protein (GFAP) with DAPI. Yellow arrow head shows a BrdU^+^ NSC (BrdU^+^SOX2^+^GFAP^+^ cell in the SGZ), pink arrow head shows a BrdU^‐^ NSC, light blue arrow heads show a BrdU^+^ progenitor (BrdU^+^SOX2^+^GFAP^‐^). Scale bar = 20 µm. **(G):** Quantification of the total number of BrdU^+^ cells in the SGZ of Chd7null and ctrl mice. **(H, I):** Quantification of the number of BrdU^+^ NSCs (H) and progenitors (I) in the SGZ of Chd7null and ctrl mice. **(J, K)** Quantification of the number of proliferating (MCM2^+^) radial GFAP^+^ SGZ NSCs (J) and MCM2^+^ GFAP^‐^ proliferating cells in the SGZ of Chd7null and ctrl mice (K). For all experiments, *n* = 3–4 animals per condition. **(L)**: Representative image of a section of the DG stained with antibodies against MCM2, GFAP and DAPI. White arrow head shows a GFAP^+^MCM2^+^ NSC, blue arrow head shows a GFAP^+^MCM2^‐^ quiescent NSC. Scale bar = 20 µm. **(M)** RT‐qPCR quantification of *Chd7* expression in cultured fetal‐derived NSCs from *Nestin‐Cre;Chd7^f/f^* (Chd7null) and Cre‐negative (control; Ctrl) embryos under growth conditions (Growth) and after incubation with media containing 50 ng/ml BMP4 (quiescence), relative to ctrl. Note *Chd7* expression is completely absent from Chd7null cells. *n* = 4 cultures. **(N):** Western blot for CHD7 on total cell lysates of cultured fetal‐derived NSCs from Chd7null (NestinCre;Chd7‐/‐) and control (Chd7+/+) cells. Cleavage and polyadenylation specificity factor 100 (CPSF100) was used as a loading control. **(O):** Quantification of the percentage of proliferating (Ki67^+^) control and Chd7null cells in vitro under growth conditions and after incubation with media containing 50 ng/ml BMP4 (quiescence). Note that Chd7null cells are refractory to quiescence induction after 72 and 96 hours in quiescence media. Both Chd7null and ctrl cells are able to return to a proliferative state when returned to growth conditions (growth media). *n* = 4 cultures. All data represented as mean ± SEM; *, *p* < 0.05; ***, *p* < 0.001 Student's *t*‐test. Abbreviations: BrdU, 5‐bromodeoxyuridine; CHD7, chromodomain–helicase‐DNA‐binding protein 7; CPSF100, cleavage and polyadenylation specific factor 2, 100 kDa subunit; DAPI, 4′,6‐diamidino‐2‐phenylindole; GFAP, glial fibrillary acidic protein; MCM2, minichromosome maintenance deficient 2 mitotin; NSC, neural stem/progenitor cell; SGZ, subgranular zone; TAM, tamoxifen.

Previous work has shown that the deletion of CHD7 from hippocampal NSCs resulted in defects in neuronal differentiation 26. This study did not examine the effects of *Chd7* deletion on the quiescent NSC population. To determine whether *Chd7* deletion affected NSC characteristics, *GLAST::CreER^T^^2^;Chd7^f/f^* and Cre‐negative control mice were injected with TAM and examined 7 days later (Fig. 3A). DG sections were immunostained for SOX2 and GFAP to identify stem (SOX2^+^GFAP^+^ SGZ cells with a radial morphology) and progenitor cells (SOX2^+^GFAP^‐^ SGZ cells) 16, ASCL1 to mark a subset of stem/progenitor cells and DCX to visualize immature neurons. The numbers of stem and progenitor cells and DCX^+^ immature neurons were not altered 7 days after TAM injection (Fig. 3B–3E), indicating that the cellular composition of the DG remained the same at this time‐point.

We next asked whether cell proliferation was affected by *Chd7* depletion. Six days after TAM administration, BrdU was injected and brains were collected 24 hours later (Fig. 3A). We observed over a twofold increase in the number of cells that had passed through S‐phase during the final 24 hours of the experiment (BrdU^+^) in the SGZ of Chd7null mice compared with controls (Fig. 3F, 3G). To identify the cell type responsible for the increased proliferation in the SGZ, sections were immunostained with antibodies against BrdU, SOX2, and GFAP to distinguish NSCs (GFAP^+^SOX2^+^ SGZ cells with a radial morphology) from progenitors (GFAP^‐^SOX2^+^ SGZ cells) 16. The number of BrdU^+^ radial NSCs was over 2.5‐fold greater in Chd7null mice compared with controls (Fig. 3H). Interestingly, the number of progenitors that had cycled was the same between Chd7null and control mice (Fig. 3I), suggesting that *Chd7* primarily regulates NSC and not progenitor proliferation. Indeed, the number of proliferating NSCs (GFAP^+^MCM2^+^ SGZ cells with a radial morphology) was increased over twofold in Chd7null mice compared with controls (Fig. 3J, 3L), whereas the number of proliferating progenitors (GFAP^‐^MCM2^+^ SGZ cells) was not altered (Fig. 3K).

To examine whether the increase in NSC number was caused by an increase in symmetric NSC divisions, we searched for the presence of two or more BrdU^+^ radial NSCs within close proximity to one another at 1, 4, and 12 weeks after Chd7 deletion. We could not detect two BrdU^+^ NSCs within close proximity to one another at any of these time points (data not shown). These results suggest that there is no dramatic shift in NSC divisions to favor symmetric cell divisions in Chd7null cells. However, a more extensive and sophisticated analysis may be required to accurately compare the propensity of Chd7null and control NSCs to undergo symmetric versus asymmetric cell divisions.

To find further evidence in support of a role for CHD7 in NSC quiescence, we used a recently described in vitro model of reversible NSC quiescence 33, 34, 35. Fetal NSCs obtained from the cortex and striatum of E16.5 *Nestin‐Cre;Chd7^f/f^* (Chd7null) and Cre‐negative embryos (control) were isolated and cultured in media containing the mitogens FGF2 and EGF 44. These cells retain multilineage differentiation capacity after prolonged expansion 31 and give a high yield of neurons when cultured under certain conditions 32. Upon removal of EGF and addition of BMP4 to the culture media, NSCs become quiescent. This quiescent state is fully reversible upon BMP4 withdrawal and EGF addition 33, 35. We confirmed that *Chd7* was expressed at high levels in control NSCs under growth conditions (Fig. 3M). *Chd7* transcripts were downregulated by approximately 68% under quiescent conditions, consistent with previous studies 26. No *Chd7* transcripts (Fig. 3M) or CHD7 protein could be detected in Chd7null cells (Fig. 3N). Control NSCs stopped proliferating and entered a quiescent state within 72 hours of transfer into quiescent culture conditions (Fig. 3O). By contrast, >10% of Chd7null NSCs were refractory to quiescent induction and remained in a proliferative state even after 96 hours of culture with BMP4 (Fig. 3O, Quiescence media). Chd7null cells retained the capacity to fully reactivate to a proliferative state (Fig. 3O, Growth media). These in vitro findings further support our identification of CHD7 as a regulator of NSC quiescence.

### Loss of *Chd7* Causes a Transient Increase in Immature Neuron Production

**Figure 4:**
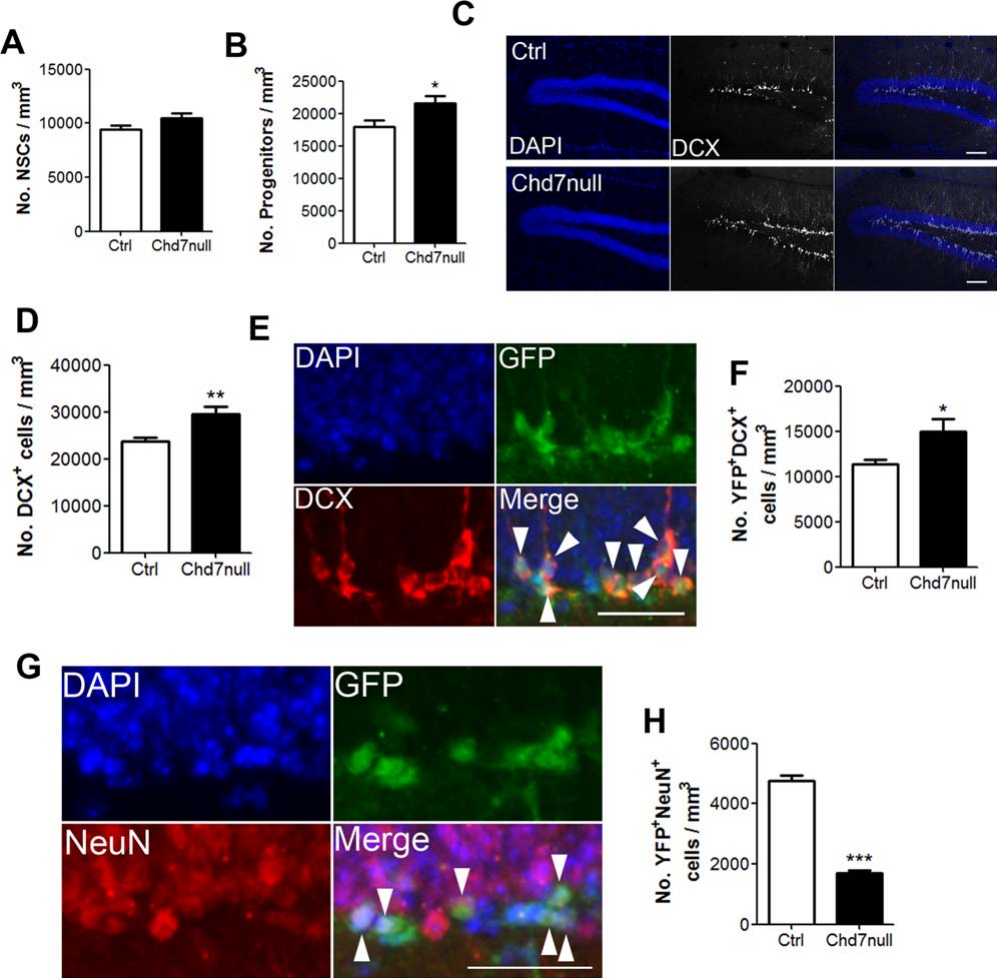
Loss of *Chd7* and neural stem/progenitor cell (NSC) quiescence results in an increase in immature neuron production. **(A, B):** Quantification of the number of radial GFAP^+^SOX2^+^ NSCs (A) and GFAP^‐^SOX2^+^ progenitor cells in the subgranular zone (SGZ) (B) of *GLAST::CreER^T2^;Chd7^f/f^* (Chd7null) and Cre‐negative control (Ctrl) mice 4 weeks after tamoxifen (TAM). **(C):** Representative image of a section of the dentate gyrus (DG) of *GLAST::CreER^T2^;Chd7^f/f^* (Chd7null) and Cre‐negative control (Ctrl) mice 4 weeks after TAM injection stained with antibodies against DCX. Scale bar = 100 µm. **(D):** Quantification of the number of DCX^+^ cells in the DG of Chd7null and ctrl mice 4 weeks after TAM injection. **(E):** Representative image of a section of the DG of *GLAST::CreER^T2^;Chd7^f/f^;RYFP/+* mice 4 weeks after TAM injection stained with antibodies against green fluorescent protein (GFP) and DCX. White arrow heads show GFP^+^DCX^+^ cells. Scale bar = 50 µm. **(F):** Quantification of the number of YFP^+^DCX^+^ newly generated immature neurons in the DG of *GLAST::CreER^T2^; RYFP/+* (control; Ctrl) and *GLAST::CreER^T2^;Chd7^f/f^;RYFP/+* (Chd7null) mice 4 weeks after TAM injection. **(G):** Representative image of a section of the DG of *GLAST::CreER^T^;RYFP/+* mice 4 weeks after TAM injection stained with antibodies against GFP and NeuN. White arrow heads show GFP^+^NeuN^+^ cells. Scale bar = 50 µm. **(H):** Quantification of the number of YFP^+^NeuN^+^ new mature neurons in the DG of *GLAST::CreER^T2^;RYFP/+* (control; Ctrl) and *GLAST::CreER^T2^;Chd7^f/f^;RYFP/+* (Chd7null) mice 4 weeks after TAM injection. *n* = 4 animals per condition. All data represented as mean ± SEM; *, *p* < 0.05; **, *p* < 0.01; ***, *p* < 0.001 Student's *t*‐test. Abbreviations: DAPI, 4′,6‐diamidino‐2‐phenylindole; DCX, doublecortin; GFP, green fluorescent protein; NSC, neural stem/progenitor cells.

Loss of NSC quiescence has been shown in some instances to lead to a transient increase in neurogenesis caused by increased conversion of stem cells to lineage restricted progenitors 15. To test whether a loss of NSC quiescence in Chd7null mice was associated with increased neurogenesis shortly after *Chd7* deletion, *GLAST::CreER^T^^2^;Chd7^f/f^* (Chd7null) and Cre‐negative control mice were examined 4 weeks after TAM injection and sections of the DG were stained with antibodies against GFAP and SOX2 to mark NSCs and progenitors and DCX to label immature neurons. Chd7null mice exhibited no significant change in the number of NSCs (Fig. 4A), and a small increase in the number of GFAP^‐^SOX2^+^ progenitors (Fig. 4B) and immature neurons in the DG compared to controls (Fig. 4C, 4D), showing that a loss of *Chd7* in NSCs results in an increase in immature neuron formation. To further show that loss of *Chd7* and loss of NSC quiescence causes a transient increase in immature neuron production, *GLAST::CreER^T^^2^;Chd7^f/f^;RYFP/+* (Chd7null) and *GLAST::CreER^T^^2^; RYFP/+* (control) mice were examined 4 weeks after TAM injection. Sections of the DG were stained with antibodies raised against green fluorescent protein (GFP) and DCX to label newly generated immature neurons (Fig. 4E). This analysis confirmed that immature neuron production is increased in Chd7null mice compared to controls (Fig. 4F). However, the number of GFP^+^NeuN^+^ newly produced neurons was decreased in Chd7null mice compared with controls (Fig. 4G, 4H), consistent with a previous study showing that CHD7 is required for NSC differentiation 26. Taken together, our data are consistent with two roles for CHD7: 1) in maintaining NSC quiescence (as reported in the present manuscript) and 2) neuronal maturation (as shown here and reported by Feng et al. 26).

### CHD7 Regulates Hippocampal Neurogenesis

**Figure 5:**
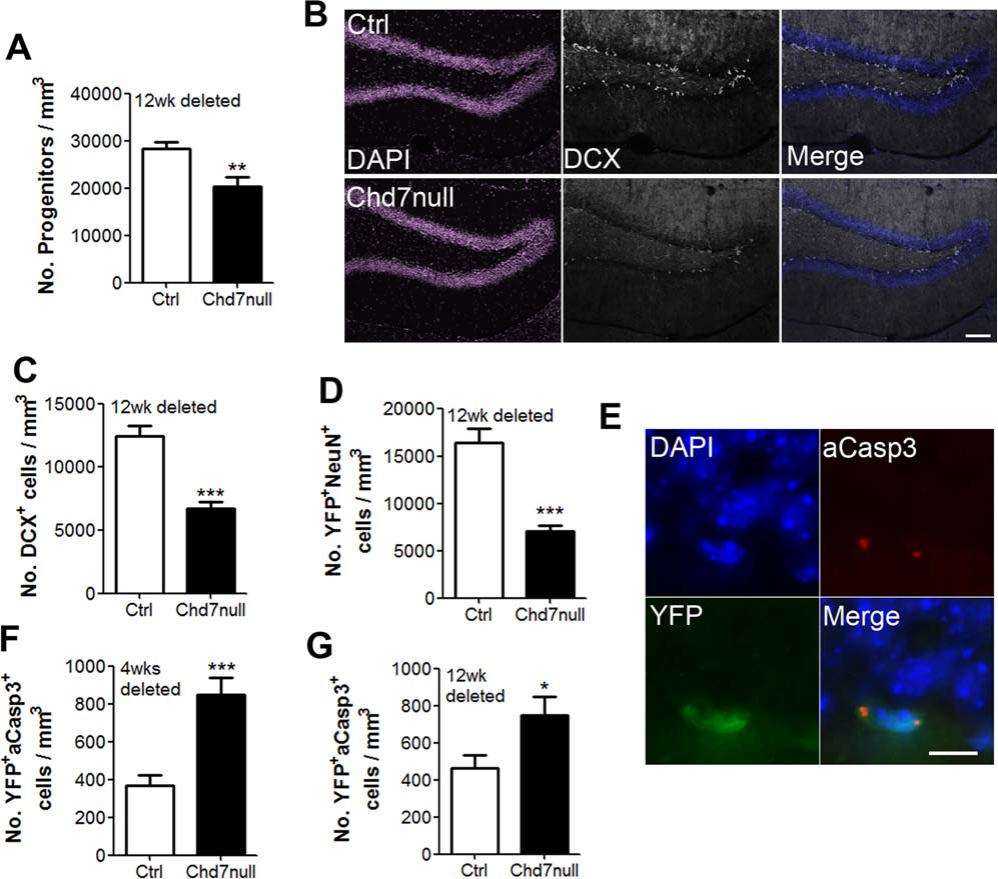
Loss of *Chd7* results in a decrease in neurogenesis and apoptosis of newly generated cells. **(A):** Quantification of the number of GFAP^‐^SOX2^+^ subgranular zone (SGZ) progenitor cells in the dentate gyrus (DG) of *GLAST::CreER^T2^;Chd7^f/f^* (Chd7null) and Cre‐negative control (Ctrl) mice 12 weeks after tamoxifen (TAM). **(B):** Representative image of a section of the DG of Chd7null and control (Ctrl) mice 12 weeks after TAM injection stained with antibodies against DCX. Scale bar = 100 µm **(C):** Quantification of the number of DCX^+^ cells in the DG of Chd7null and ctrl mice 12 weeks after TAM injection. *n* = 4 animals / condition. **(D):** Quantification of the number of YFP^+^NeuN^+^ cells in the DG of *GLAST::CreER^T2^;RYFP/+* (control; Ctrl) and *GLAST::CreER^T2^;Chd7^f/f^;RYFP/+* (Chd7null) mice 12 weeks after TAM injection **(E):** Representative image of a section of the DG of *GLAST::CreER^T2^;Chd7^f/f^;RYFP/+* mice 4 weeks after TAM injection stained with antibodies against green fluorescent protein (GFP) (YFP) and cleaved caspase‐3 (aCaps3) showing an apoptotic non‐neural stem/progenitor cells (NSC) (nonradial) cell. Scale bar = 10 µm. **(F, G):** Quantification of the number of YFP^+^aCasp3^+^ (apoptotic) newly generated cells in *GLAST::CreER^T2^;RYFP/+* (Ctrl) and *GLAST::CreER^T2^;Chd7^f/f^;RYFP/+* (Chd7null) mice 4 weeks (F) and 12 weeks (G) after TAM injection. All data represented as mean ± SEM; *, *p* < 0.05; **, *p* < 0.001; ***, *p* < 0.001 Student's *t*‐test. Abbreviations: DAPI, 4′,6‐diamidino‐2‐phenylindole; DCX, doublecortin; NSC, neural stem/progenitor cells; YFP, yellow fluorescent protein.

We have shown that loss of *Chd7* causes a loss of NSC quiescence followed by a transient increase in progenitor number and immature neuron production soon after deletion. To determine the longer‐term effect of *Chd7* deletion on neurogenesis, adult *GLAST::CreER^T^^2^;Chd7^f/f^* (Chd7null) and Cre‐negative control mice (control) mice were examined 12 weeks after TAM injection. In agreement with Feng et al., Chd7null mice exhibited a significant decrease in the number of GFAP^‐^SOX2^+^ progenitors (Fig. 5A) and immature (DCX^+^) neurons (Fig. 5B, 5C) 26. Furthermore, staining adult hippocampal sections of *GLAST::CreER^T^^2^;RYFP/+* (control) and *GLAST::CreER^T^^2^;Chd7^f/f^;RYFP/+* (Chd7null) mice 12 weeks after TAM injection with antibodies to GFP and NeuN showed an impaired ability of Chd7null mice to generate new granule neurons (Fig. 5D). To determine the cause of the reduction in progenitor and immature neuron number in Chd7null mice 12 weeks after deletion, we stained hippocampal sections of adult *GLAST::CreER^T^^2^;RYFP/+* (control) and *GLAST::CreER^T^^2^;Chd7^f/f^;RYFP/+* (Chd7null) 4 and 12 weeks after TAM injection with antibodies to GFP and cleaved caspase‐3 (aCasp3), a marker of apoptosis. Chd7null mice displayed an increased number of apoptotic cells. Based on morphology, apoptosis was present in the population of newly generated GFP^+^ progenitor (type 2 and 3) cells, and not in NSCs with radial morphology (Fig. 5E–5G). These data confirm that *Chd7* is required for normal levels of neurogenesis and the survival of newborn neural progenitors in the adult DG. Collectively, our data suggest a model whereby the loss of NSC quiescence results in the premature conversion of stem cells into progenitors, which fail to fully mature and are eliminated by apoptosis.

**Figure 6:**
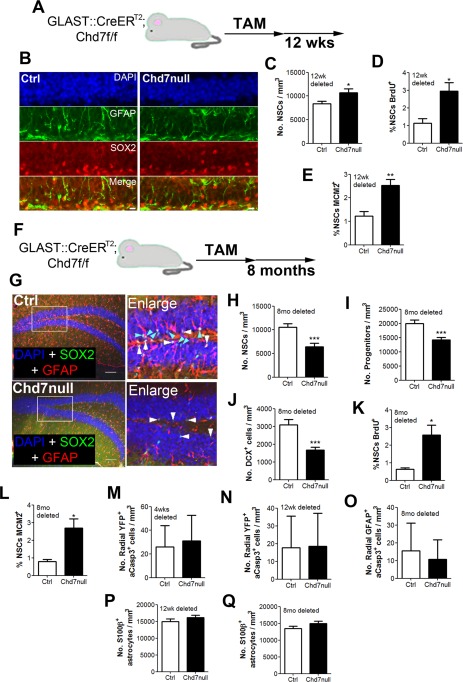
Deletion of *Chd7* from adult NSCs results in the depletion of the stem cell pool. **(A):** Schematic diagram of the experimental strategy to delete *Chd7* from adult NSCs for 12 weeks. **(B):** Representative image of a section of the dentate gyrus (DG) of *GLAST::CreER^T2^;Chd7^f/f^* mice treated with tamoxifen (TAM) (Chd7null) and Cre‐negative controls (Ctrl) stained with antibodies against glial fibrillary acidic protein (GFAP) and SOX2 with DAPI 12 weeks after *Chd7* deletion. Scale bar = 10 µm **(C):** Quantification of the number of GFAP^+^SOX2^+^ radial NSCs in the subgranular zone (SGZ) of ctrl and Chd7null mice 12 weeks after TAM. **(D, E):** Quantification of the number of BrdU^+^ NSCs (BrdU^+^SOX2^+^GFAP^+^; D) and MCM2^+^GFAP^+^ radial NSCs (E) in the SGZ of Chd7null and ctrl mice. **(F):** Schematic diagram of the experimental strategy to delete *Chd7* from adult NSCs for 8 months. **(G):** Representative image of a section of the DG of Cre‐negative controls and *GLAST::CreER^T2^;Chd7^f/f^* mice treated with TAM (Chd7null) and stained with antibodies against GFAP and SOX2 with DAPI 8 months after *Chd7* deletion. Note a reduction in radial GFAP processes through the granular layer and a reduction in SOX2^+^ cells in the SGZ in Chd7null sections. Boxed areas, enlarged in the ‘Enlarge’ panel, show radial GFAP^+^SOX2^+^ NSCs (light blue arrowheads) and GFAP^‐^SOX2^+^ progenitors (white arrowheads). **(H, I):** Quantification of the number of GFAP^+^SOX2^+^ radial NSCs (H) and GFAP^‐^SOX2^+^ progenitors (I) in the SGZ of ctrl and Chd7null mice 8 months after TAM administration. **(J):** Quantification of the number of DCX^+^ immature neurons in the DG of ctrl and Chd7null mice 8 months after TAM injection. **(K, L):** Quantification of the number of BrdU^+^ NSCs (BrdU^+^SOX2^+^GFAP^+^; K) and MCM2^+^GFAP^+^ radial NSCs (L) in the SGZ of Chd7null and ctrl mice 8 months after TAM injection. **(M, N):** Quantification of the number of SGZ YFP^+^GFAP^+^ radial NSCs that are apoptotic (aCasp3^+^) in the DG of *GLAST::CreER^T2^; RYFP/+* (Ctrl) and *GLAST::CreER^T2^;Chd7^f/f^;RYFP/+* (Chd7null) mice 4 weeks **(M)** and 12 weeks **(N)** after TAM injection. Note the very low numbers of apoptotic NSCs at both time points. **(O):** Quantification of the number of apoptotic (aCasp3^+^) radial GFAP^+^ NSCs in the SGZ of Cre‐negative controls and *GLAST::CreER^T2^;Chd7^f/f^* mice 8 months after TAM injection. Note the low number of apoptotic NSCs. **(P, Q)**: Quantification of the number of S100β^+^ mature astrocytes in the granular layer, SGZ and hilus of Cre‐negative controls and *GLAST::CreER^T2^;Chd7^f/f^* mice 12 weeks (P) and 8 months (Q) after TAM injection. For all experiments, *n* = 3–4 animals per condition. All data represented as mean ± SEM; *, *p* < 0.05; **, *p* < 0.01; ***, *p* < 0.001 Student's t test. Abbreviations: BrdU, 5‐bromodeoxyuridine; CHD7, chromodomain–helicase‐DNA‐binding protein 7; DCX, doublecortin; GFAP, glial fibrillary acidic protein; NSC, neural stem/progenitor cells; TAM, tamoxifen; YFP, yellow fluorescent protein.

### Deletion of *Chd7* and Loss of NSC Quiescence Results in the Depletion of the NSC Pool

Adult stem cell quiescence is essential for the preservation of a functional stem cell pool throughout life 10, 13, 15. To determine whether *Chd7* deletion affected hippocampal stem cell number, adult *GLAST::CreER^T^^2^;Chd7^f/f^* (Chd7null) and Cre‐negative control mice were examined 12 weeks after TAM injection (Fig. 6A). DG sections were stained with antibodies against the stem cell markers GFAP and SOX2 and the number of GFAP^+^SOX2^+^ radial SGZ NSCs were counted (Fig. 6B). Interestingly, at this time point, Chd7null mice displayed an increase in the number of SGZ NSCs (Fig. 6C). To determine if NSCs were more proliferative in Chd7null mice, Chd7null and control mice were injected with BrdU 24 hours before examination, 12 weeks after TAM injection. The number of BrdU^+^ radial NSCs was almost threefold greater in Chd7null mice compared to controls (Fig. 6D) and the number of MCM2^+^ proliferating radial NSCs was over twofold greater (Fig. 6E), consistent with the increased proliferation of this population observed at earlier time points (Fig. 3H, 3J).

Sustained NSC proliferation has been shown in other studies to be associated with the premature depletion of the stem cell pool 13, 19. To determine whether *Chd7* deletion has a similar effect, Chd7null and control mice were examined 8 months after TAM injection (Fig. 6F). Indeed, Chd7null mice displayed a significant depletion of NSCs, and a reduction in progenitor cell numbers and immature neurons (Fig. 6G–6J). Furthermore, the remaining NSCs in Chd7null mice were more proliferative than in controls (Fig. 6K, 6L) suggesting that NSC depletion is likely to continue during further ageing. Taken together, our data are consistent with other studies in which loss of NSC quiescence is linked to NSC depletion 13, 19. To determine if the loss of Chd7 and NSC quiescence led to increased apoptosis of NSCs, hippocampal sections of adult *GLAST::CreER^T^^2^;RYFP/+* (control) and *GLAST::CreER^T^^2^;Chd7^f/f^; RYFP/+* (Chd7null) were stained with antibodies against GFP and cleaved caspase‐3 and the number of apoptotic radial YFP^+^ NSCs were counted at 4 weeks and 12 weeks after TAM injection. Apoptotic NSCs were very rare in control and Chd7null mice (Fig. 6M, 6N), indicating that depletion of the NSC pool is unlikely to be caused by NSC apoptosis. Furthermore, the number of apoptotic radial SGZ GFAP^+^ cells was also very rare 8 months after TAM injection in *GLAST:: CreER^T^^2^;Chd7^f/f^* (Chd7null) and Cre‐negative control mice (Fig. 6O).

To rule out the possibility that the NSC pool is depleted through differentiation into astrocytes, adult *GLAST:: CreER^T^^2^;Chd7^f/f^* (Chd7null) and Cre‐negative control mice were stained with antibodies against the mature astrocyte marker S100β 12 weeks and 8 months after TAM injection and the number of astrocytes in the granular layer and hilus of the DG counted. *Chd7* deletion did not change the number of mature astrocytes present in the DG at both timepoints (Fig. 6P, 6Q), suggesting that the stem cell pool is not depleted due to differentiation of NSCs into astrocytes. Taken together, our results are suggestive of a model whereby the NSC pool is depleted primarily through the loss of NSC quiescence, resulting in the premature conversion of stem cells into progenitors, which fail to fully differentiate (Fig. 4G, 4H) and are eliminated by apoptosis (Fig. 5E–5G).

### CHD7 Regulates the Expression of Genes Involved in Cell Cycle Progression and Notch Signaling

**Figure 7:**
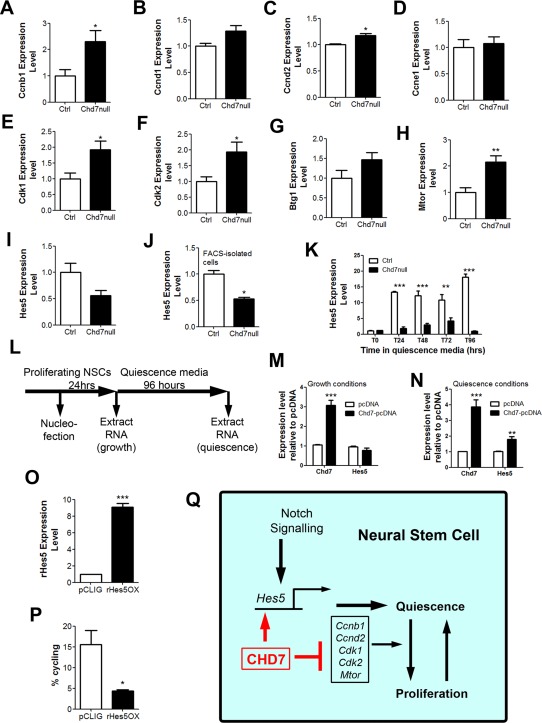
CHD7 regulates NSC quiescence through the expression of cell cycle genes. **(A–I):** RT‐qPCR expression level of *Ccnb1* (A), *Ccnd1* (B), *Ccnd2* (C), *Ccne1* (D), *Cdk1* (E), *Cdk2* (F), *Btg1* (G), *Mtor* (H), and *Hes5* **(I)** from microdissected dentate gyrus (DG) of *GLAST:: CreER^T2^;Chd7^f/f^* (Chd7null) and Cre‐negative control (Ctrl) mice 3 weeks after the tamoxifen (TAM) injection. Note a general increase in the expression of many cell cycle genes. *n* = 4 animals (biological replicates) per condition. **(J):** RT‐qPCR expression level of *Hes5* in YFP^+^Dapi^‐^ FACS‐isolated NSCs from *GLAST::CreER^T2^;Chd7^f/f^; RYFP/+* (Chd7null) and *GLAST::CreER^T2^;RYFP/+* (Ctrl) mice 7 days after the final TAM injection. *n* = a minimum of 1000 cells from 4 animals per condition. **(K):** RT‐qPCR expression level of *Hes5* from cultured fetal‐derived NSCs from *Nestin‐Cre;Chd7^f/f^* (Chd7null) and Cre‐negative (control; Ctrl) embryos under growth conditions (T0) and after incubation with media containing 50 ng/ml BMP4 (quiescence) for 24 (T24), 48 (T48), 72 (T72), and 96 (T96) hours, relative to ctrl. *n* = four reactions. **(L):** Schematic diagram of the experimental strategy to nucleofect Chd7‐pcDNA 36 or pcDNA empty vector control into control cells and extract the RNA from nucleofected cells under growth and quiescence conditions. **(M, N):** RT‐qPCR expression level of human *Chd7* (which may also recognize mouse *Chd7*) and mouse *Hes5* in Chd7‐pcDNA and pcDNA control nucleofected cells after 24 hours in growth media (M) or 24 hours in growth media followed by 96 hours in quiescence media (N). **(O, P):** RT‐qPCR expression of rat *Hes5* (which may also recognize mouse *Hes5*) (O) and quantification of cycling (Ki67^+^) cells (P) in rHes5‐pCLIG and pCLIG control nucleofected Chd7null cells after 24 hours in growth media followed by 24 hours in quiescence media. All data represented as mean ± SEM; *, *p* < 0.05; **, *p* < 0.01; ***, *p* < 0.001 Student's t test. **(Q):** Schematic diagram of the role of CHD7 in adult hippocampal NSC quiescence. CHD7 maintains NSC quiescence by directly promoting the expression of *Hes5* and inhibiting the expression of positive regulators of cell cycle progression to maintain stem cell quiescence. Abbreviations: CHD7, chromodomain–helicase‐DNA‐binding protein 7; NSC, neural stem/progenitor cells.

The findings presented so far identified CHD7 as an essential regulator of NSC quiescence and showed that even a relatively subtle loss of NSC quiescence can result in the depletion of the NSC pool over time 13, 19. As previous studies have implicated cell cycle regulators and the Notch signaling pathway in NSC quiescence and since the loss of cyclin‐dependent kinases and Notch signaling components result in depletion of the NSC pool 13, 16, 17, 18, 19, we hypothesized that CHD7 might function upstream of these factors. To test this hypothesis, RNA was extracted from the dentate gyri of Chd7null and control mice and the expression of genes involved in positive and negative cell cycle regulation assayed by RT‐qPCR. The expression of several genes encoding cyclins and cyclin‐dependent kinases, including *Ccnb1*, *Ccnd2*, *Cdk1*, and *Cdk2* were significantly upregulated in Chd7null samples (Fig. 7A–7F). By contrast, the expression of the cell cycle inhibitor, *Btg1*, was not affected by the loss of *Chd7* (Fig. 7G) 45, 46. We also observed a significant increase in the expression of *Mtor*, a key regulator of adult NSC quiescence and proliferation 47, 48, in the Chd7null DG (Fig. 7H). Engelen et al. recently reported the genome‐wide distribution of CHD7 protein on chromatin in NSCs 27. This study detected significant enrichment of the *Mtor* promoter after CHD7 chromatin immunoprecipitation, thereby identifying *Mtor* as a likely direct CHD7 target 27.

Reduced Notch signaling in adult NSCs results in the loss of NSC quiescence and the eventual depletion of the stem cell pool 15, 30, 49. The *Hes5* gene is a critical downstream effector of Notch signaling in adult hippocampal NSCs 16, 18, 43, 50. Data from Engelen et al. suggested that CHD7 is recruited to the *Hes5* gene promoter in NSCs. Together with the demonstration that *Hes5* expression is downregulated in *Chd7^+/^^‐^* NSCs, these findings imply a direct role for CHD7 in regulating *Hes5* expression 27. To determine whether CHD7 is required for normal levels of *Hes5* expression in the adult DG, we quantified the expression of *Hes5*. Microdissected DG from Chd7null mice displayed a tendency for reduced *Hes5* expression (Fig. 7I). As *Hes5*‐expressing NSCs represent only a small fraction of the total cells in the DG, we enriched for NSCs by FACS‐sorting GFP^+^ cells from *GLAST::CreER^T^^2^;Chd7^f/f^; RYFP/+* and *GLAST::CreER^T^^2^;RYFP/+* mice 7 days after TAM injection. *Hes5* expression was significantly reduced in Chd7null cells compared to controls (Fig. 7J), identifying CHD7 as a critical regulator of *Hes5* expression.

To further examine the regulation of *Hes5* by CHD7 we analyzed the dynamics of *Hes5* expression in NSCs during quiescence induction 33, 34. Control NSCs rapidly upregulated *Hes5* expression upon exposure to quiescence medium (Fig. 7K). By contrast, Chd7null cells were unable to fully upregulate *Hes5* expression (Fig. 7K), indicating that CHD7 is required for *Hes5* expression during quiescence. To find evidence that CHD7 directly regulates *Hes5* expression, a *pcDNA::hChd7* expression construct was transfected into NSCs. RNA was extracted from cells after incubation in growth media for 24 hours, or quiescence media for an additional 96 hours (Fig. 7L). Under growth conditions, the expression of *Chd7* increased 3‐fold in *pcDNA::hChd7*‐transfected cells compared to *pcDNA*‐transfected controls but the expression of *Hes5* was unaffected (Fig. 7M), suggesting that under conditions that force proliferation of NSCs, CHD7 expression is not sufficient to induce *Hes5* expression. However, under quiescence conditions, CHD7 over‐expression resulted in a 1.8‐fold upregulation of *Hes5* expression (Fig. 7N). These results are consistent with *Hes5* being a direct and immediate target of CHD7 under conditions that promote NSC quiescence.

To investigate the functional requirement of *Hes5* expression in the context of CHD7 deficiency, we transfected Chd7null cells with a *pCLIG*::*rHes5* expression construct (rHes5OX) or empty vector control. Cells were cultured in growth media for 24 hours after transfection and then incubated in quiescence media for a further 24 hours. RNA was extracted or cells fixed and immunostained for Ki67 to determine proliferation. r*Hes5* expression (Fig. 7O) was sufficient to drive Chd7null cells into quiescence (Fig. 7P), showing that *Hes5* functions downstream of CHD7 to induce NSC quiescence.

In summary, these data link pathways with established roles in NSC quiescence to CHD7 (Fig. 7Q).

## Discussion

Several studies have suggested that the loss of stem cell quiescence in adult neurogenic niches can result in the premature depletion of the NSC pool 13, 15, 16, 17, 18, 19, 20. Preservation of a quiescent state depends on the inhibition of genes involved in cell cycle progression and the maintenance of genes encoding cell cycle inhibitors. In the present study we have uncovered a role for CHD7 in the maintenance of NSC quiescence and differentiation of neuronal progenitors in the adult hippocampus 26. The loss of CHD7 in adult hippocampal stem cells is associated with the increased expression of cyclins and cyclin‐dependent kinases involved in cell cycle progression (Fig. 6L). Furthermore, the Notch effector gene, *Hes5* is downregulated upon CHD7 deletion. We propose that this combination of gene expression changes together are responsible for the loss of NSC quiescence in the absence of CHD7.

At present, it is unclear precisely how the regulation of CHD7 expression is related to NSC quiescence. CHD7 levels are clearly highest in activated, proliferative hippocampal neuronal progenitors, both in vivo and in vitro (this study and Feng et al. 26). We propose that CHD7 expression is upregulated as quiescent NSCs become activated and enter the cell cycle and that this CHD7 upregulation is necessary for daughter cells to return to quiescence to maintain the NSC pool. When Chd7null cells enter the cell cycle, our data suggest that they would be unable to fully upregulate *Hes5* expression. A failure to induce Hes5 expression might render these cells unable to respond properly to Notch signals in the niche leading to their reduced ability to return to quiescence. Future studies to identify NSC dynamics in the intact stem cell niche are required to test this model 3.

A recent study by Feng et al. showed that CHD7 is required for neuronal differentiation by remodeling the promoters of *Sox4* and *Sox11* to create an open chromatin state 26. SOX4 and SOX11 have been shown previously to play an essential role in adult neurogenesis by binding directly to the *Dcx* promoter and inducing its expression 51. Interestingly, we find that the number of DCX^+^ cells is not reduced shortly after loss of *Chd7*, and is even increased 4 weeks after *Chd7* deletion (Fig. 3B, 3L). These findings argue against a simple model whereby CHD7 regulates neurogenesis primarily through a SOX4/SOX11/DCX mechanism. It will be of significant interest to identify the SOX4 and SOX11‐regulated target genes that mediate CHD7‐dependent neuronal differentiation.

A recent study by Micucci et al. showed that CHD7 is required for the self renewal of adult NSCs derived from the subventricular zone (SVZ) of the lateral ventricles in vitro 25. Furthermore, deletion of *Chd7* from embryonic NSCs resulted in a large decrease in SVZ proliferation in the perinatal SVZ 25. Micucci et al. could not detect any changes in the expression of cell cycle regulators in the SVZ of *Chd7*‐deficient mice, in contrast to our findings in the hippocampus 25. These differences may be reflected by the timing of TAM‐induced deletion of *Chd7* (embryonic and adolescent compared to adult), or may reflect differences in hippocampal and olfactory bulb neurogenesis. Interestingly, Chd7null NSCs in vitro can be passaged for many months (data not shown), suggesting that in vitro, NSC self‐renewal potential is not dramatically reduced in the absence of CHD7.

Our data, together with those showing CHD7 recruitment to the *Hes5* promotor 27, identify *Hes5* as a key CHD7 target required for NSC quiescence. Notch signaling is highly active in adult NSCs and its persistence in adult neurogenic niches has been shown to be essential for maintaining NSC quiescence 16, 18, 43. Loss of Notch signaling has been shown to cause the activation of NSCs, followed by a transient increase in immature neuron formation and the eventual depletion of the stem cell pool through replicative exhaustion 16, 17, 18. The similarities of these phenotypes to those described here upon *Chd7* deletion, lend support to the notion that CHD7 mediates at least some of its effects through *Hes5*. Of note, Notch signaling is key in maintaining quiescence in other somatic stem cell systems, including in adult skeletal muscle satellite cells 49, 52, 53 and pancreatic progenitors 54. It is tempting to speculate that CHD proteins may have similar roles in the maintenance of Notch signaling and quiescence in other adult stem cell populations. Another key regulator of adult NSC quiescence is BMP signaling, that can convert rapidly proliferating NSCs in culture into a state of reversible quiescence 33, 35. Interestingly, a recent study has revealed an interaction between CHD7 and the downstream BMP effectors SMAD1/5/8 in embryonic cardiomyocytes 55. It would be interesting to examine the possibility that CHD7 regulates NSC quiescence through an effect on BMP signaling.

Various critical roles for epigenetic regulators in stem cell quiescence are beginning to emerge 56, 57, 58. For example, the chromatin remodeling enzyme CHD4 is required for hematopoietic stem cell (HSC) maintenance, through regulating the expression of receptors that mediate interactions between HSCs and their niche, and repressing *Ccnd2* that drive HSC differentiation 59. The deletion of CHD4 in HSC populations resulted in the loss of HSC quiescence and skewed their differentiation potential to generate mainly erythroid cells 59. Taken together with our data, these findings imply central roles for chromatin remodeling factors in the maintenance of a quiescent state in somatic stem cell populations.

We have shown that the NSC and progenitor cell pool is reduced 8 months after deletion of *Chd7*. Feng et al. have shown that exercise can rescue the neurogenesis defects in CHD7 mutant mice 26. The data presented here suggest that interventions that enhance neurogenesis may only be of temporary benefit as the *Chd7*‐deficient NSC pool will eventually become depleted due to the failure to maintain NSC quiescence, leaving few NSCs and daughter cells to respond to exercise‐induced neurogenesis. Therefore, treatments that promote NSC maintenance in addition to those that enhance neurogenesis will be required to maintain sufficient levels of neuronal production throughout life 60.

## Conclusion

Hippocampal NSCs are mostly quiescent, a property critical for the maintenance of the stem cell pool throughout life. Loss of NSC quiescence was shown previously to cause a premature conversion of stem cells to lineage‐restricted progenitors and an eventual loss of neurogenesis and the NSC pool 15. Here, CHD7 is identified as a critical regulator of NSC quiescence in the adult hippocampus. We propose that CHD7 controls NSC quiescence through regulating the expression of multiple genes involved in cell fate and proliferation. We have identified *Hes5* as one key CHD7 target gene through which CHD7 maintains NSC quiescence.

## Author Contributions

K.M.J. and M.A.B.: conception and design, collection and assembly of data, data analysis and interpretation, manuscript writing, final approval of manuscript; N.S., J.P.R., and C.L.A.: collection and assembly of data, data analysis and interpretation, final approval of manuscript; P.J.S.: conception and design, manuscript writing, final approval of manuscript.

## Disclosure of Potential Conflicts of Interest

The authors indicate no potential conflicts of interest.

## Supporting information

Supporting Table 1
