# Supplementary material for: CHD7 Maintains Neural Stem Cell Quiescence and Prevents Premature Stem Cell Depletion in the Adult Hippocampus
Source: Stem Cells. 2014 Dec 18;33(1):196–210. doi: 10.1002/stem.1822 (PMC5952591; doi:10.1002/stem.1822)
Supplement: Supplementary file 1 — Supporting Table 1 [file STEM-33-196-s001.docx]

Supplementary Table 1: PCR primer sequences (to amplify mouse sequences unless stated otherwise)

| **Target** | **Forward primer (5'-3')** | **Reverse primer (5'-3')** |
| --- | --- | --- |
| *Gapdh* | AGGTCGGTGTGAACGGATTTG | TGTAGACCATGTAGTTGAGGTCA |
| *Hes5* | AGTCCCAAGGAGAAAAACCGA | GCTGTGTTTCAGGTAGCTGAC |
| r*Hes5* | CAAACTGGAGAAGGCCGACATC | CACGAGTAACCCTCGCTGTAG |
| *Chd7* | TCACCAGCCTTGGGCACAACTC | TAGCTGAGCGTTCTGTGCGCTG |
| h*Chd7* | TTCACCTCCACACCCTCATCAC | AGTCATATCCGGCACTGGTTTC |
| *Pax6* | GAGAAGAGAAGAGAACTGAGGAA | ATTGGCTGGTAGACACTGGTA |
| *Ccnb1* | AAGGTGCCTGTGTGTGAACC | GTCAGCCCCATCATCTGCG |
| *Ccnd1* | CAACTTCCTCTCCTGCTACCG | ACTCCAGAAGGGCTTCAATCTG |
| *Ccnd2* | AAAGCTGTGCATTTACACCGAC | GCGAAGGATGTGCTCAATGAAG |
| *Ccne1* | GTGGCTCCGACCTTTCAGTC | CACAGTCTTGTCAATCTTGGCA |
| *Cdk1* | AGAAGGTACTTACGGTGTGGT | GAGAGATTTCCCGAATTGCAGT |
| *Cdk2* | CCTGCTTATCAATGCAGAGGG | GTGCTGGGTACACACTAGGTG |
| *Cdkn1b* | ACACTTGATCACTGAAGCCTCG | CTGCACACAGACAGTCAAATGG |
| *Mtor* | ACCGGCACACATTTGAAGAAG | CTCGTTGAGGATCAGCAAGG |
| *Nestin* | AGAGTCAGATCGCTCAGATCC | GCAGAGTCCTGTATGTAGCCAC |
| *Btg1* | AAGTTCCTCCGCACCAAGGG | GATGCGAATACAACGGTAACCTG |
